# An improved beetle antennae search path planning algorithm for vehicles

**DOI:** 10.1371/journal.pone.0274646

**Published:** 2022-09-15

**Authors:** Qing Liang, Huike Zhou, Yafang Yin, Wei Xiong

**Affiliations:** 1 Xi’an University of Posts and Telecommunications, School of Electronic Engineering, Xi’an, Shaanxi Province, China; 2 Xi’an University of Posts and Telecommunications, School of Automation, Xi’an, Shaanxi Province, China; Beijing Institute of Technology, CHINA

## Abstract

With the development of society, the application of mobile robots in industry and life is increasingly extensive, and the local path planning of mobile robots in unknown environments is a problem that needs to be solved. Aiming at the problem that the traditional beetle antennae search (BAS) algorithm can easily fall into local optimum and the optimization accuracy is low, we propose an improved beetle antennae search. It introduces a map safety threshold, the addition of virtual target points, and the smoothing of the path. Map safety threshold means extra space with obstacles at all times, improving path reliability by avoiding collisions. Adding virtual target points reduces situations where the vehicle gets stuck in local optima. The B-spline smoothing path reduces the original path’s straight turns to improve the path’s robustness. The effectiveness and superiority of the algorithm are verified by comparing and testing the existing path planning algorithms through simulation in different environments.

## 1. Introduction

In recent years, mobile robots have played an essential role in people’s daily life. Mobile robots undertake many tasks that people need to complete [1]. Overall, the research field of mobile robots can be divided into the following sections: navigation and localization [2], environment perception and modeling [3], and multi-robot coordination [4]. Robots have strong application prospects in the industrial field and medical services, special explosion-proof, field exploration, deep-sea exploration, household services, and other areas. These fields require robots to have more efficient path planning, and these fields have attracted the attention of many researchers.

Navigation and positioning can generally divide into sub-directions: positioning, environment establishment, and path planning. Among these research directions, the collision-free path planning task of mobile robots is an important direction. The collision-free path planning task can summarize as a collision-free path from the starting point to the target point according to certain criteria in an environment with obstacles. According to the map information, it can divide into global and local path planning. In current daily life of people, mobile robots do not always carry out global path planning in an environment with known obstacles, and most robots use unknown environments in their work tasks. Therefore, local path planning is more practical than global path planning. This paper mainly studies the problem of vehicle local path planning.

In previous studies, researchers mainly considered using different algorithms to solve the path planning problem of mobile robots without collision: for example, the A* algorithm [5, 6], RRT algorithm [7, 8], Dijkstra algorithm [9, 10], and artificial potential field method (APF) [11, 12] and so on.

Li proposed an improved Dijkstra algorithm, and the improved algorithm can obtain the shortest distance to the target point. However, when the scale of the grid map is large, the Dijkstra algorithm’s search efficiency is slow, and the problem is still not effectively improved [13]. Nie uses the Dijkstra algorithm for initial path planning and then improves the ant colony algorithm (ACO) to optimize the initial path. There are also shortcomings that the local optimum has not completely solved [14]. Zhang and others introduced radar threat function and three-dimensional bidirectional sector-shaped multi-layer variable-step search strategy in the traditional A* algorithm to meet the waypoint accuracy and algorithm search efficiency [15]. Hou uses an improved Q-Learning algorithm combined with artificial potential field path planning to improve planning efficiency [16].

Miao proposed that the angular guiding factor and the obstacle removal factor are introduced into the transition probability of the ACO algorithm [17]. Fu proposed an algorithm combining APF-ACO. The inflection point optimization algorithm is used to reduce the number and length of inflection points in the path, and the curve fitting algorithm is used to optimize the path [18]. Wang combines polynomial curves and artificial algorithms to enable motion planning after vehicle impact and adjust the vehicle’s trajectory and yaw motion to achieve obstacle avoidance function [19]. Zhang proposed to generate quadratic programming-based trajectory clustering in the case of invalid driving conditions and assign different timestamps to each waypoint through time sampling [20]. Jiang proposed an intelligent algorithm combining the ant colony algorithm and the BAS algorithm, using ACO to generate the initial path and then the BAS algorithm to orient the search to ensure the stability of its pathfinding [21]. Wu also proposed the Obstacle Avoidance BAS algorithm for robot path planning [22]. The authors mentioned above have made improvements in path planning and path smoothing. However, there are still problems such as easy falling into local optimization, long search time of the algorithm, and poor path smoothing effect.

To solve the above problems, we introduced an improved BAS-based virtual target point algorithm called virtual beetle antennae search (VBAS). We applied the improved algorithm to the path planning problem of vehicles, and the algorithm has a low time complexity while satisfying the safety and collision-free obstacles. The remainder of this paper is organized as follows: in section 2, the problem definition that this paper needs to solve describe in detail. In section 3, the VBAS algorithm, introduces two aspects of algorithm flow and improvement. In section 4 evaluates the performance of its improved VBAS algorithm through simulation and comparison with other algorithms. In section 5, the full text and final outlook summarize. The main contributions of this paper are as follows.

1. The virtual target point adds to the traditional BAS algorithm, and a new intelligent optimization algorithm VBAS proposes, which improves the ability of the vehicle to fall into the local extreme value.
2. The proposed VBAS algorithm can avoid obstacles in the local path planning problem and has the advantage of fast planning.
3. The algorithm’s effectiveness verifies in the vehicle application, and the vehicle’s superiority in path planning under the requirement of fast obstacle avoidance is proven by comparing it with the existing algorithm.

## 2. Problem description

This paper mainly solves the problem of local path planning. Specifically, it is necessary to solve the problem of planning a collision-free path from the starting point to the target point in a static environment with unknown obstacle information. The map is preset, the static obstacles are also present, and the vehicle only detects the obstacle information within the current range. The initial point and the target point are given in advance. This relationship is embodied in a 0–1 matrix as follows.

$$M_{i,j}=\{\begin{array}{l} 1,\;\mathrm{if}\;M_{i,j}\notin O\\ 0,\;\mathrm{if}\;M_{i,j}\in O \end{array}$$ (1)

where *M*_*i*,*j*_ is the element map, and *O* is the obstacle area. The proposed algorithm is suitable for the path planning problem with an objective function. The objective function with constraints on the path can generally express Formula 2.

$$g(x)=x-x_{tar},\;x\notin O$$ (2)

where *g*(⋅) is the function to be optimized, *x*∈*R*^*k*^ the current point coordinate, *x*_*tar*_∈*R*^*k*^ the target point coordinate, and *k* is the dimension of the planning space. For the planned path to be effective, the function to be optimized needs to be as small as possible.

**Fig 1:**
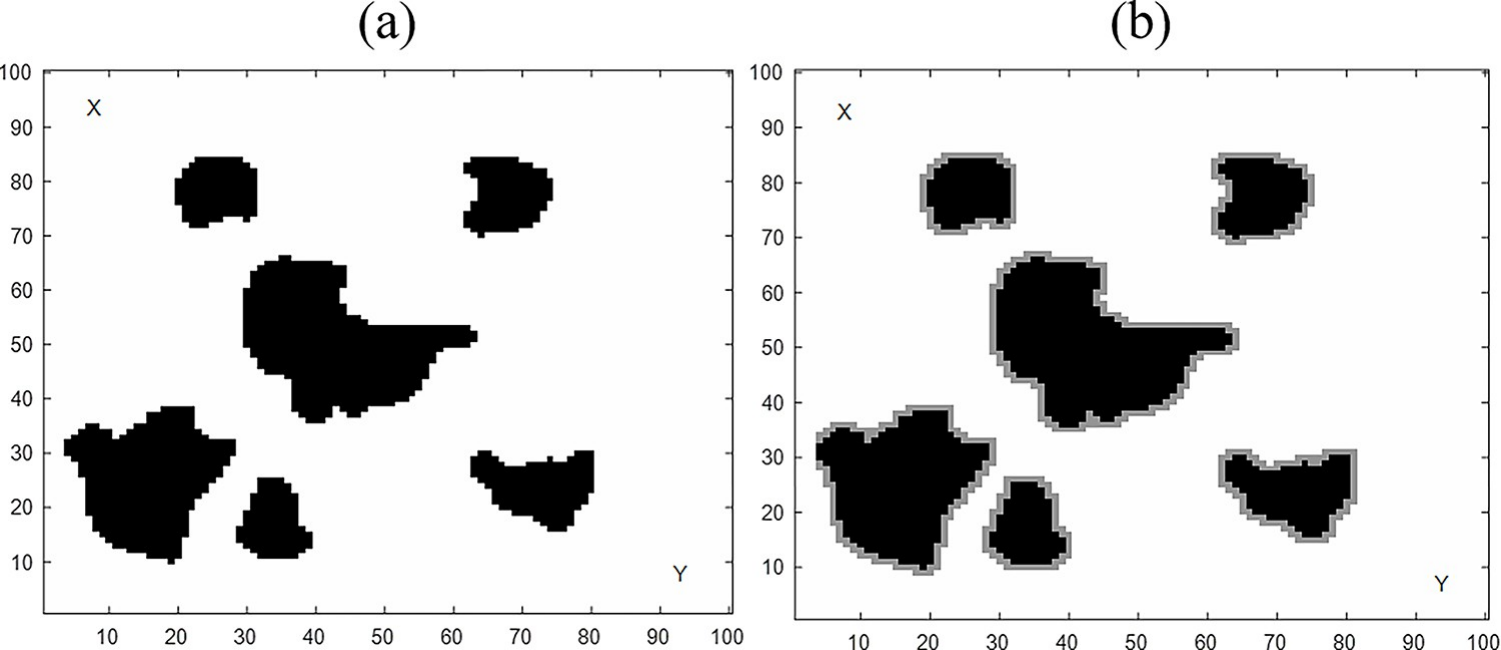
Two-dimensional simulation environment map. (a) Real Environment Map. (b) Safety threshold environment map.

The path generated by the algorithm may be very close to the obstacle, and if the path is used directly as a planned path by the vehicle, the vehicle will have a high risk of colliding with the obstacle. Therefore, maintaining an appropriate distance from obstacles must be considered during path planning. In this paper, the concept of a safety threshold is proposed. Since the algorithms in this paper are all based on the beetle antennae algorithm, the initial moving step of the BAS is used as its safety threshold. Fig 1A is a 100×100 pixel map of the simulated environment. The black grid represents the obstacle area, which is 0 in Formula 1, and the rest of the area is a non-obstacle area, which is 1 in Formula 1. Fig 1B shows this paper to construct a global grid map integrated with the safety level, express the distance between obstacles in a more detailed manner, establish a safety threshold, and solve the problem of nodes approaching obstacles in the vehicle’s planned path.

When all the waypoints in the set of waypoints planned by the VBAS algorithm are not in the obstacle area, and within the safety threshold, this planning is considered valid. When this path is directly connected, it is very tortuous, increasing the vehicle’s energy consumption and losing its moving speed. Therefore, the generated path needs to be smooth to ensure that it can use for robot movement. While ensuring the feasibility of the path, planning time is also an important attribute. The faster the planning time, the more practical the algorithm is. Based on the description of the above problem, the goal we need to achieve is to plan a smoother collision-free path from the starting point to the goal point for the vehicle as quickly as possible.

## 3. VBAS algorithm

The traditional BAS is an algorithm inspired by the foraging principle of beetle antennae search. The main purpose of this paper is to plan a collision-free path from the source point to the target point on the map. The distribution of the objective function is equivalent to the distribution of food smells in the space. The beetle flies in a certain direction according to its perception, and the scent information collected by the antennas is the basis for the next optimization. According to the different scent concentrations of the left and right whiskers, the running trajectory of the next point judges until the current point coincides with the target point and the algorithm iteration ends. At this time, the vehicle finds the maximum point of the global smell, which is the end of the path planning [23, 24]. When the antenna touches the obstacle, a virtual target point is added in front of the other side of the antennae to guide the vehicle to avoid the obstacle. When the vehicle coincides with the target point, at the end of its iterative process, at this time, the B-spline method performs to smooth the path, and the processed path set is output.

**Fig 2:**
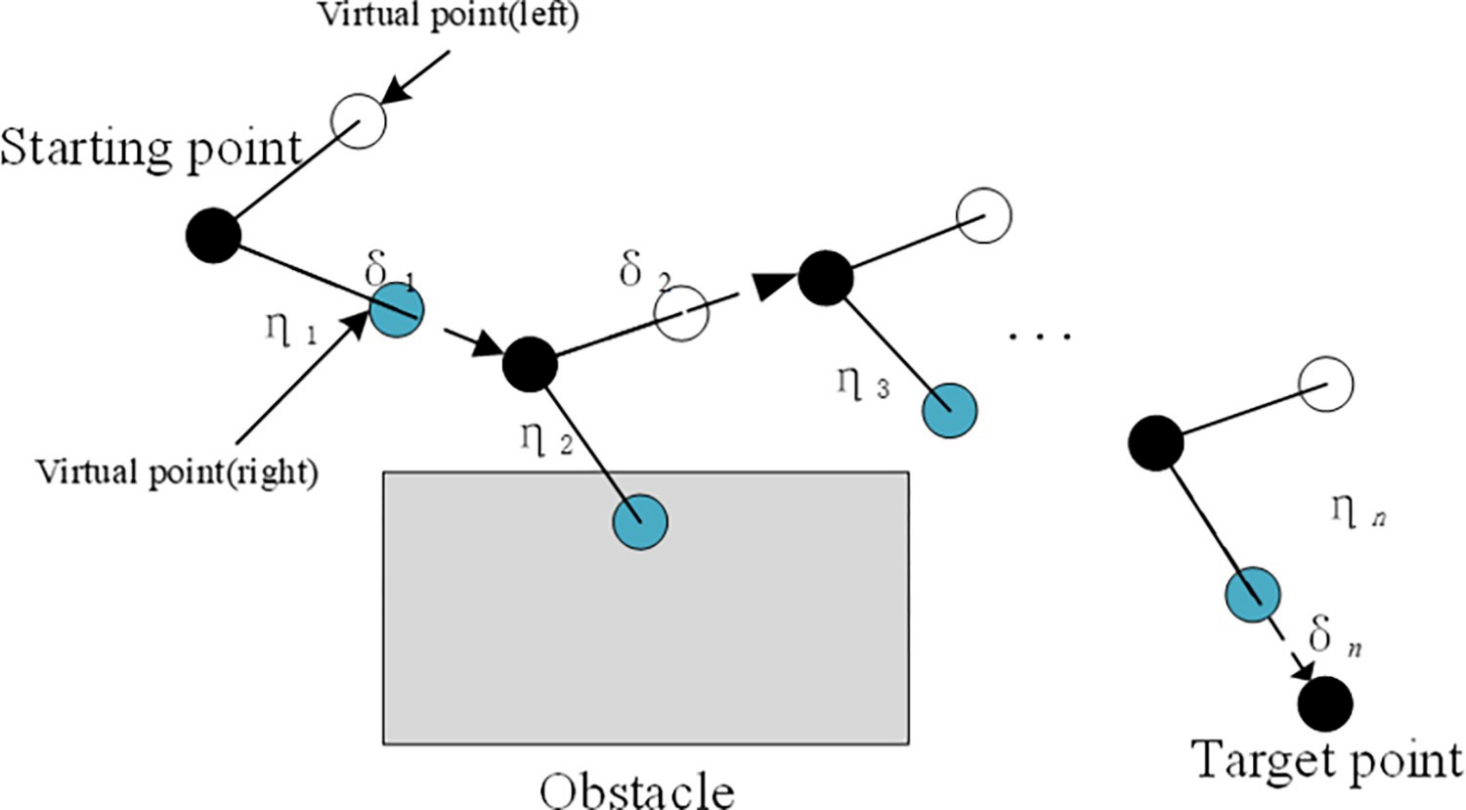
Pathfinding process of the vehicle.

Fig 2 shows the vehicle’s path planning visualization process from the starting point to the target point. The black dots represent the robot’s travel to the current position, and the two small dots represent the generated virtual target points. The virtual target point of the left antenna is white, and the right antenna is blue. The virtual target point is blue, and the black rectangle represents the obstacle area, the *η*_*i*_ represents current virtual target point range, and *i* represents the number of iterations.

The whole algorithm can divide into the following three parts: virtual target point and candidate point generation, virtual target point and candidate point selection, and final path generation.


$$\vec{d}=\frac{\mathrm{rands}\;(k,1)}{{\left\|\mathrm{rands}\;(k,1)\right\|}_{2}}$$ (3)

The rands (.) function is a random generation function and *k* is the data dimension, representing the planning space’s dimension in this paper *k* = 2. After the direction vector $\vec{d}$ obtains, the coordinates of two candidate points *x*_*l*_∈R^*k*^ and *x*_*r*_∈R^*k*^ two virtual target points *x*_*vl*_∈R^*k*^, *x*_*vr*_∈R^*k*^ are generated according to the detection distance Formulas 4 and 5.

$$\begin{array}{l} x_{l}=x+\vec{d}\delta^{t}\\ x_{vl}=x+\lambda \vec{d}\delta^{t} \end{array}$$ (4)

$$\begin{array}{l} x_{r}=x-\vec{d}\delta^{t}\\ x_{vr}=x-\lambda \vec{d}\delta^{t} \end{array}$$ (5)

where *x*∈R^k^ is the current location, *t* is the current iteration number, *λ* is the weight of the virtual target point of VBAS, which changes the size of the environment, and *δ*^*t*^ is the beetle antennae search area, which is calculated by the following Formula 6.


$$\delta^{t}=\eta \delta^{t-1}+\alpha$$ (6)

Among them *η* is the attenuation rate of the long beetle detection distance and *α* is the attenuation increase amount, which adjusts according to the size of the environment. Adjusting *η* and *α* can improve the obstacle avoidance ability and the ability to stay away from the local minima of the beetle antennae search algorithm.

### 3.1 Virtual target point selection strategy

This part aims to determine the coordinates of the next path point according to the candidate point selection in Section 3.1 and obtain the coordinates of the two candidate points and the virtual target point according to Formulas 4 and 5. The VBAS algorithm m is suitable for path planning problems with specific target points. This paper sets an objective function *f*(⋅):R^k^→R, which is essentially a function to be optimized. The next waypoint is determined based on the value of the objective function. The function to be optimized is defined below in Formula 7.

$$f(x)=\{\begin{array}{c} {\left(x-x_{tar}\right)}^{2},\;\mathrm{if}\;x\geq c,\\ {\left(x-x_{obs}\right)}^{2},\;\mathrm{if}\;x<c, \end{array}$$ (7)

where *x* is the current point coordinate, *x*_*tar*_ is the target point, *x*_*obs*_ is the obstacle with the closest d-direction vector distance to the current vehicle, and *c* is the safety threshold. When the virtual point is smaller than the obstacle threshold, switch to the function to be optimized. In this paper, the smaller the objective function value is, the closer the target function is to the target point, so the purpose is to minimize the objective function. However, if the objective function is minimized throughout the process, it may cause the vehicle to be unable to avoid obstacles.

**Algorithm** 1 VBAS algorithm for collision-free path planning of vehicle

**Input**: Map with obstacles *M*, points x_sta_ and x_tar_, the maximum number of iterations

**Output**: The path point array *Q*, the path length *L*, number of iterations i

1: Initialize the basic parameters of VBAS such as, *λ*, *δ*^*t*^, *η*, *α*, *c*

2: **repeat**

3: Generate random **d** by Formula 3

4: Calculate *x*_r_, *x*_*l*_, *x*_*vr*_ and *x*_*vl*_ by Formulas 4 and 5

5: Calculate *f*(*x*_*r*_), *f*(*x*_*l*_), *f*(*x*_*vr*_) and *f*(*x*_*vl*_) by Formula 6

6: If the *x*_*vr*_ or *x*_*vl*_ side is included in the obstacle

Generate virtual target points on the other side of *x*_*vr*_ or *x*_*vl*_

Change the target point to a virtual target point

Generate *x*_*nex*_ by Formula 8 and save *x*_*nex*_ to Q

Change the virtual target point to the target point

7: If there are no obstacles in either *x*_*vr*_ or *x*_*vl*_

Generate *x*_*nex*_ by Formula 8 and save *x*_*nex*_ to Q

8: Path oscillation is greater than the threshold

Delete oscillation path

9: **until** *x*_*nex*_ = *x*_*tar*_

10: Smooth (*Q*)

11: Calculate *L* based on Q

12: **return** *Q*, *L*, *i*

The whole objective optimization function process can divide into two states, and one is in the search process in which the virtual objective point does not touch the obstacle temporarily. The other is the search process in which the virtual target point touches the obstacle. The corresponding candidate, and point selection strategies will also be different. When the virtual target point does not touch the obstacle, the corresponding target point is the path target point. When one side of the antenna touches the obstacle, the virtual target point of the beetle on the other side will work to guide the candidate point to move towards the virtual target point. If there are no collision obstacles on both antennas, the target point switches to the path target point. This paper introduces a virtual target point, and the relevant formulas for generating the next coordinate point are as follows to realize the two-state switching.

$$x_{nex}=\{\begin{array}{l} x-\delta^{t}\vec{b}\mathrm{sign}(f(x_{r})-f(x_{l})),\;\mathrm{if}x\geq c,\\ x+\delta^{t}\vec{b}\mathrm{sign}(f(x_{vr})-f(x_{vl})),\;\mathrm{if}x<c, \end{array}$$ (8)

where *x*_*nex*_ is the coordinate of the next path point. The *f*(*x*_*vr*_), *f*(*x*_*vl*_) are objective functions between the current point and the obstacle. The *f*(*x*_*r*_), *f*(*x*_*l*_) are virtual objective functions between the current point and the target point.

$$\mathrm{sign}(x)=\{\begin{array}{l} 1,\;x>0,\\ 0,\;x=0,\\ -1,\;x<0, \end{array}$$ (9)

where the sign(⋅) is the symbolic function.

### 3.2 Judging the shock process

Since the virtual target point is close to the current position, the path oscillation problem will inevitably occur. This paper proposes to judge whether the current path set oscillates and how to remove the path set oscillation. When the average Euclidean distance between the current position and the first n points in the path set is less than the threshold, it considers that an oscillation phenomenon occurs in the path set. The path from the first n points to the current position is deleted.

$$\mathrm{mean}{\left(Q_{g}-x_{\mathrm{nex}}\right)}^{2}<c$$ (10)

where *Q*_*g*_ represents the *g* points, the *g* is adjusted according to the map size in this paper *g* = 15, and mean represents the average value.

### 3.3 Path smoothing optimization

**Fig 3:**
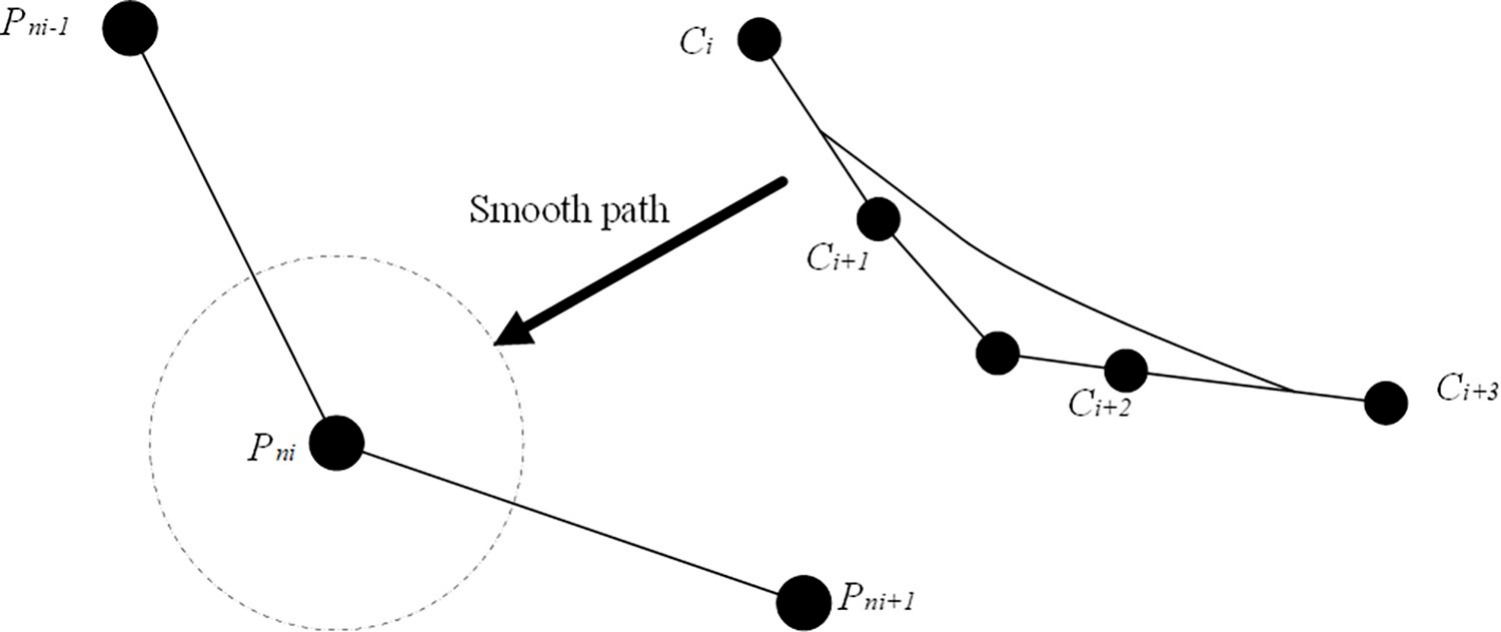
Smooth path process.

This part aims to generate a collision-free smooth path for the vehicle. Since the waypoints have been obtained in Sections 3.1 and 3.2, this part directly connects the various waypoints. The path needs to be smooth because the directly connected path points are too tortuous. Schoenberg proposed the B-spline method in 1946. In path planning, the B-spline method is a commonly used path smoothing [22, 25, 26]. The B-spline method has the following characteristics: The B-spline curve contains the convex polygon of its control polyline. Because of this characteristic, the B-spline method can modify the effect of a local path without changing the shape of the entire path. The formula of the B-spline curve method is as follows:

$$F(u)=\sum_{i=0}^{n-1}B_{i,p}(u)C_{i}$$ (11)

where n is the number of control points, *C*_*i*_ is the coordinate of the control point, and *B*_*i*,*p*_(*u*) is the spline basis function of the control point *C*_*i*_. Fig 3 shows the smooth path process.

Fig 3 shows the four control points *C*_*i*_, *C*_*i*+1_, *C*_*i*+2_, *C*_*i*+3_ and the smooth inflection point *n*_*i*_, the adjacent coordinates, and three points are the center points of the three adjacent key inflection points, which are iteratively solved by Formulas 12 and 13.

$$B_{i,0}(u)=\{\begin{array}{ll} 1, & \mathrm{if}\;u_{i}\leq u<u_{i+1}\\ 0, & \mathrm{if}\;\mathrm{otherwise} \end{array}$$ (12)

$$B_{i,d}(u)=\frac{u-u_{i}}{u_{i+q}-u_{i}}B_{i,d-1}(u)+\frac{u_{i+p+1}-u}{u_{i+p+1}-u_{i}}B_{i+1,d-1}(u)$$ (13)

where *u* is the knot node, the number of which is N, determined by order of the spline function *d* = 3 and the number of control points *n*.


N=d+n (14)

In this paper, to ensure that the smooth curve is more in line with the constraints of the kinematics and dynamics of the vehicle, the order of the sampling bar function *d* = 3 and the number of control points *n* = 4, and the set of knots node sequence *U* = [*u*_0_,*u*_1_,*u*_2_,*u*_3_,*u*_4_,*u*_5_,*u*_6_] control points can be obtained.

Finally, through the control point *C*_*i*_ corresponding to the spline basis function *B*_*i*,*d*_(*u*), the B-Spline can be obtained as:

$$F(u)=[C_{i}C_{i+1}C_{i+2}C_{i+3}][\begin{array}{c} B_{i,3}(u)\\ B_{i+1,3}(u)\\ B_{i+2,3}(u)\\ B_{i+3,3}(u) \end{array}]$$ (15)

The ${\left[B_{i,3}(u)B_{i+1,3}(u)\;B_{i+2,3}(u)\;B_{i+3,3}(u)\right]}^{T}$ is the set of control points. After connecting the set of paths, the complete path planning process obtains.

**Fig 4:**
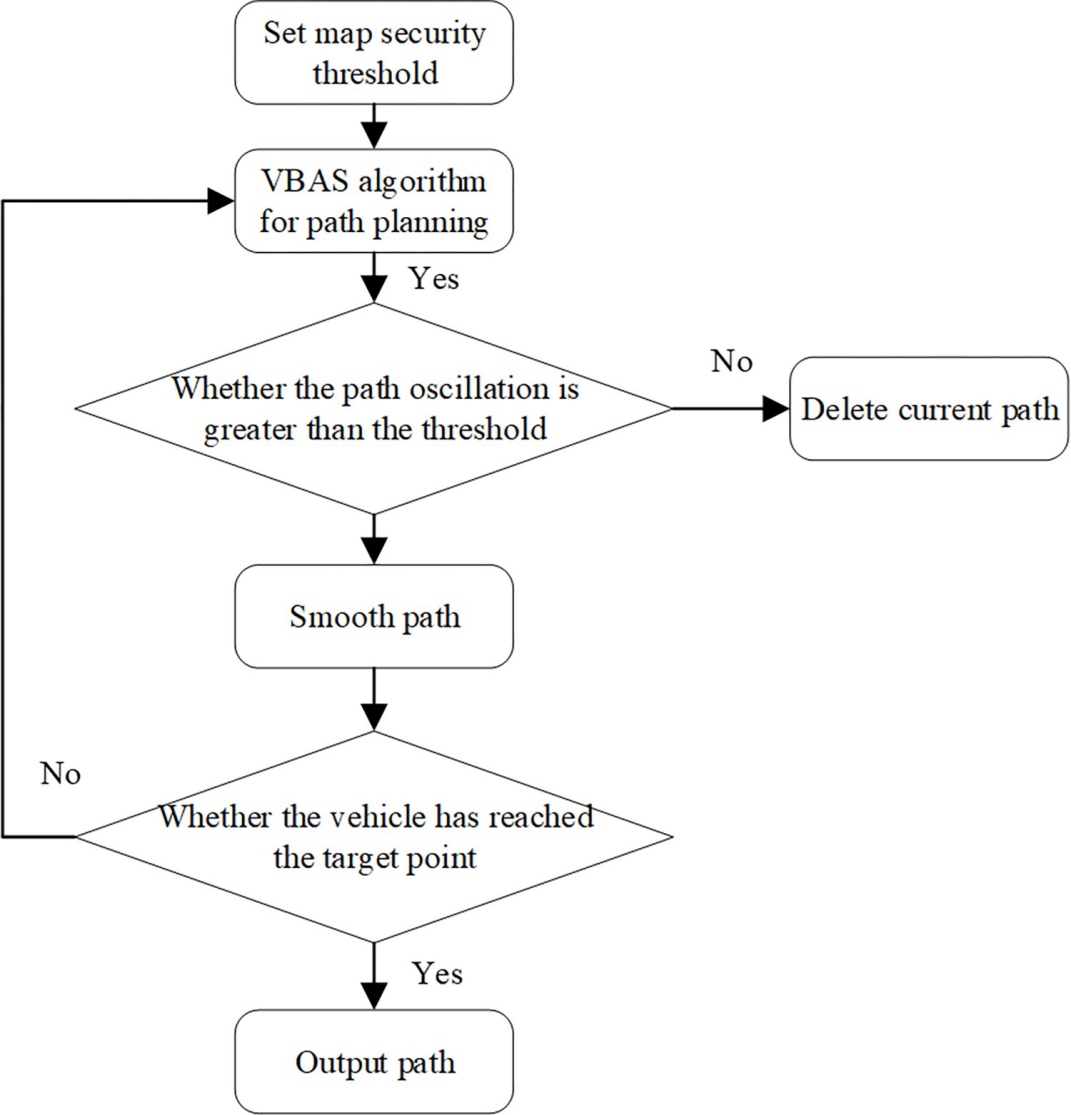
Path planning process.

In Algorithm 1, the main flow of the VBAS algorithm describes in detail, and its pseudocode provide. Fig 4 shows the overall path planning process.

## 4. Algorithm simulation and comparison

The algorithm proposed in this paper is suitable for path planning problems with objective functions. This section aims to compare and verify the effectiveness of the VBAS algorithm through a series of simulations and comparisons with other algorithms. The following algorithms verify the algorithm’s effectiveness by averaging the results of multiple simulations. Think of the vehicle as a particle. In Section 4.1, four simulation results are selected for in-depth analysis, proving the robustness and generality of the VBAS algorithm. In Section 4.2, the VBAS algorithm compares with other algorithms. By comparison, the superiority of the algorithm is proven.

All simulations in this paper are implemented on MATLAB and use the same parameters. The size of the simulated map is 450×450 pixels. On this map, the final parameters take the following values: the step size is 0.5. The initial radius of the beetle is 0.5. The initial radius of the virtual target point is 1. The step size decay rate *η* = 0.95, the step size decay increment *α* = 0.005, the decay rate, decay increment and step value of the beetle and the virtual target point are the same. The weight of the virtual target point of the beetle *λ* = 5.

### 4.1 Illustrative examples

The purpose of this section is to verify the effectiveness of the VBAS algorithm through multiple simulation results. The simulation maps shown in this section can divide into single and multiple obstacles, according to the number of obstacles. They divide into regular obstacles according to their type and irregular obstacles. This paper presents the simulation results using single and multiple obstacles.

#### 4.1.1 Single obstacle

**Fig 5:**
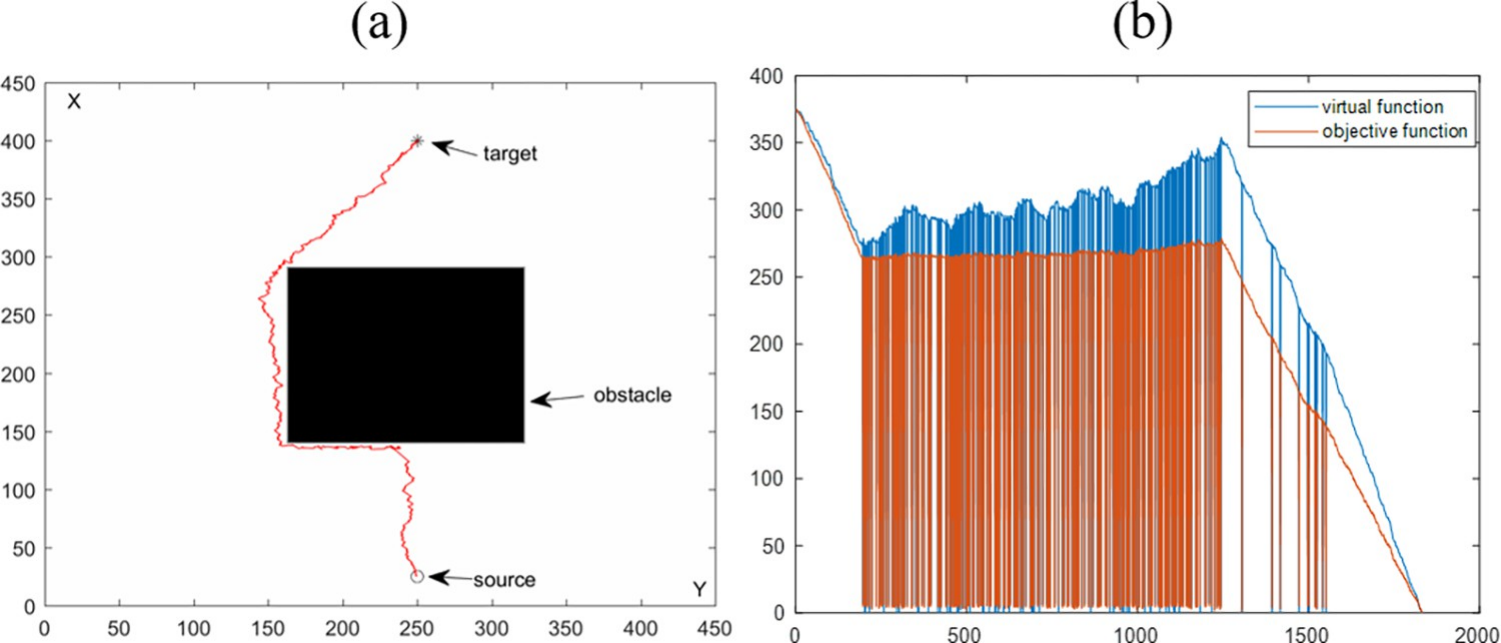
Path planning results are synthesized by the VBAS algorithm to avoid a single regular obstacle. (a) Motion results in a 2D plane. (b) Relationship between function and number of iterations.

Fig 5 shows the simulation results of a mobile robot for obstacle avoidance with a single regular obstacle, and Fig 5A is the path planning result. Fig 5B shows the virtual function value of the objective function and the virtual target point’s left antenna distance from the current point as a function of the number of iterations. In Fig 5A, the results prove that the algorithm proposed in this paper can plan a collision-free path in a single regular obstacle environment.

**Fig 6:**
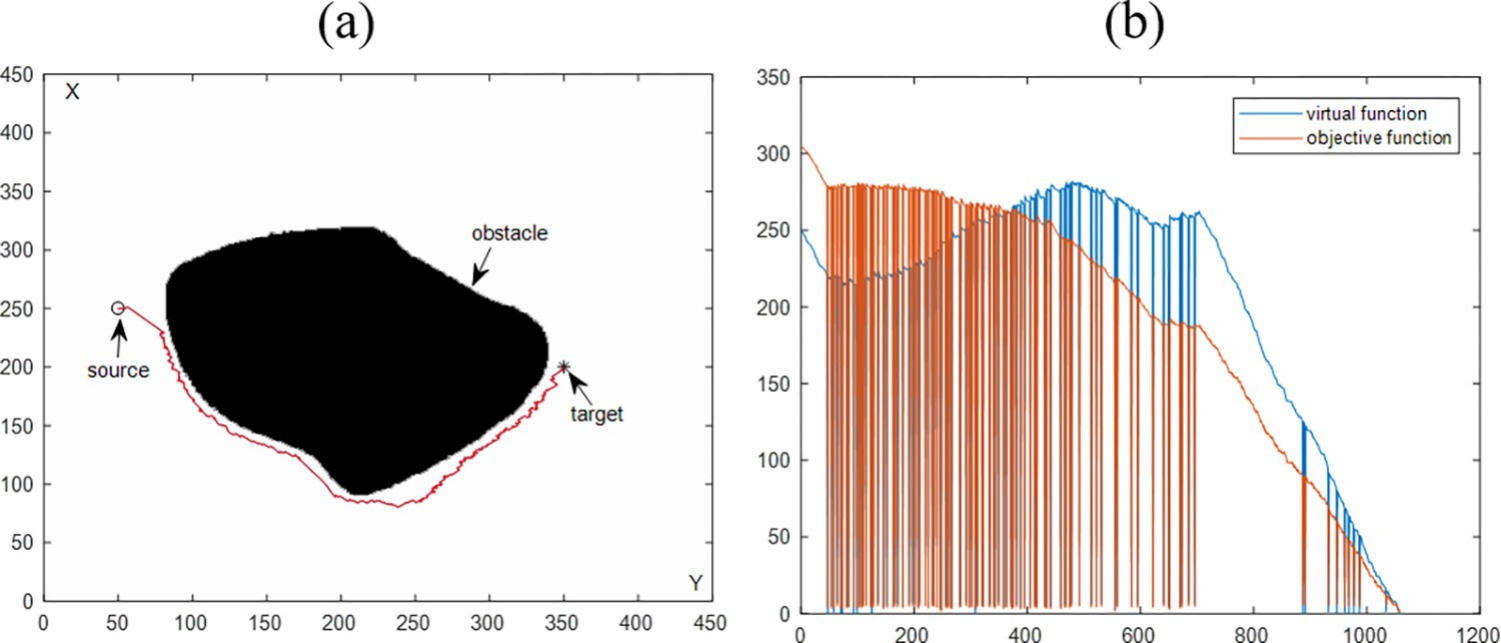
Path planning results are synthesized by the VBAS algorithm to avoid a single irregular obstacle. (a) Motion results in a 2D plane. (b) Relationship between function and number of iterations.

Fig 6 shows the simulation results of obstacle avoidance for a single irregular obstacle. The difference between the obstacles in Figs 5 and 6 is that one is a regular obstacle, and the other is an irregular obstacle. Fig 6A is the simulation planning result of a single irregular obstacle, and Fig 6B is the changing trend of the objective function value with the number of iterations. Combining the two simulation results, it can conclude that for irregular obstacles, the algorithm also can avoid obstacles.

#### 4.1.2 Multiple obstacles

**Fig 7:**
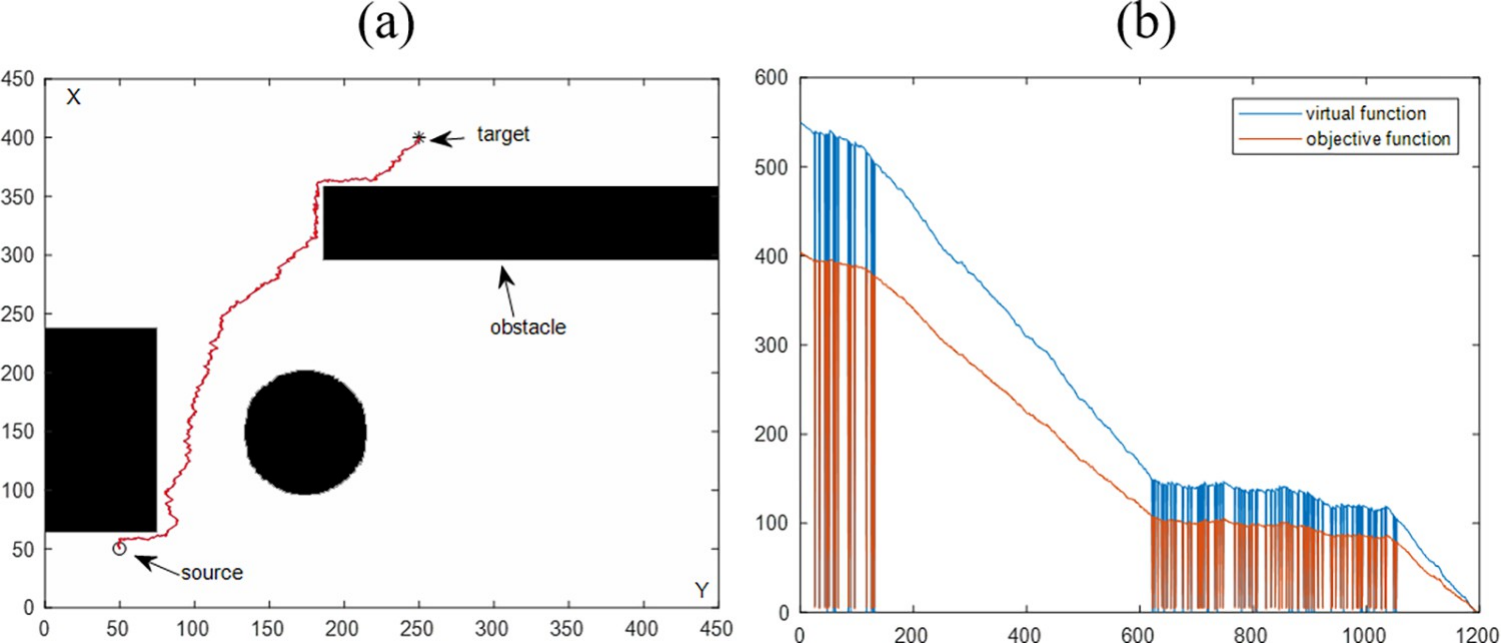
Path planning results are synthesized by the VBAS algorithm to avoid multiple regular obstacles. (a) Motion results in a 2D plane. (b) Relationship between function and number of iterations.

**Fig 8:**
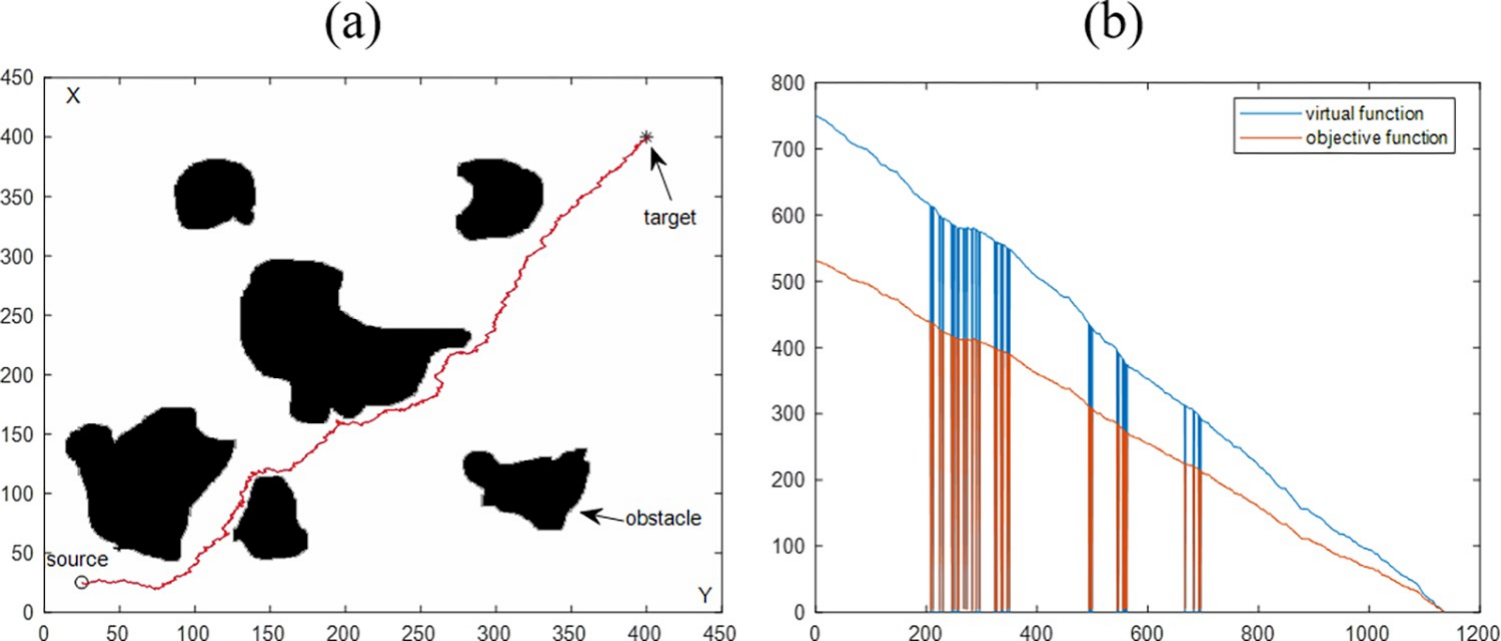
Path planning results are synthesized by the VBAS algorithm to avoid multiple irregular obstacles. (a) Motion results in a 2D plane. (b) Relationship between function and number of iterations.

Figs 7 and 8 are the simulation results of multiple regular and irregular obstacles. Similar to the single-obstacle simulation, Fig 7A visually shows the visualization results of path planning in a multi-obstacle environment. Fig 8B shows the objective function value versus the number of iterations. Combining the above two simulation results, it can conclude that the proposed algorithm has the obstacle avoidance ability for multiple obstacles. By comprehensively analyzing the four simulation results in Section 4.1, it can be proved that VBAS can plan collision-free paths in various obstacle environments.

#### 4.1.3 Statistics of simulation results

The purpose of this section is to evaluate the performance of the VBAS algorithm with the four simulation results in Sections 4.1.1 and 4.1.2. Table 1 is the statistics of the above four simulation results. P represents the path length, and n is the number of iterations. Each of the above simulations was run 100 times with the same parameters, and the average running time was obtained to evaluate the VBAS algorithm. In Fig 5A, when the current point is close to the obstacle, the oscillation is obvious, which is caused by the switching between the virtual target point and the target point. After the path generates, the path oscillation judgment will perform. If the oscillation exceeds the threshold, it will delete the current path subset. The steep drop of the function curve in Fig 5B is because of the target point switch, and the target function drops sharply when the target point switch to the virtual target point. The objective function rises sharply when the virtual target point is switched to the target point.

**Table 1:** Path planning performance of VBAS algorithm under different types of obstacles.

Type of obstacle	source	target	p/m	t/s	i
Single regular	25,250	400,250	941.172	0.356	1865
Single irregular	250,50	200,350	678.323	0.139	1210
Multiple regular	50,50	400,250	763.124	0.587	1195
Multiple irregular	25,25	400,400	1114.316	0.322	1313

### 4.2 Comparison

This section conducts extensive comparisons with existing algorithms to further demonstrate the superiority of the VBAS algorithm proposed in this paper. To verify the algorithm’s effectiveness in many aspects, we simply compare the VBAS algorithm with other algorithms. Running time and path length are compared as evaluation metrics. The running time reflects the real-time nature of the algorithm. The shorter the runtime, the more likely it is to achieve real-time path planning. On the premise of successful obstacle avoidance, the path length is as short as possible. The overall comparison results are shown in Table 2. It can be seen from Table 2 that the VBAS algorithm generally performs in terms of path length but has obvious advantages in running time.

**Table 2:** Comparing the path planning of the VBAS algorithm and APF under different types of obstacles.

Type of obstacle	p-VBAS/m	p-APF/m	t-VBAS/s	t-APF/s
Single regular	922.189	1016.227	0.359	1.149
Single irregular	671.331	823.697	0.254	1.193
Multiple regular	860.56	958.109	0.198	2.326
Multiple irregular	1123.149	1272.002	0.205	1.22

#### 4.2.1 Comparison of APF algorithms

**Fig 9:**
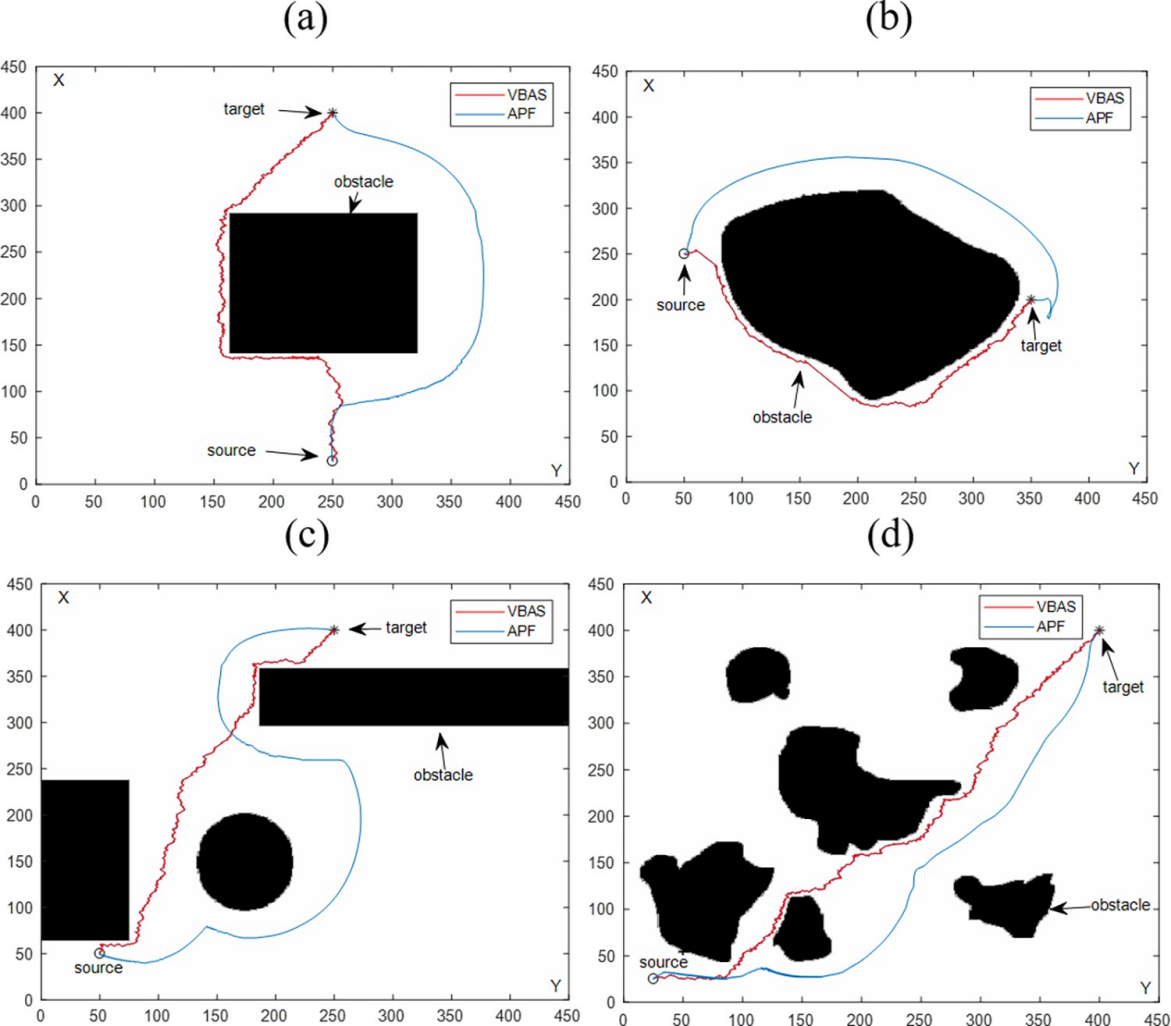
Comparison of obstacle avoidance results between VBAS and APF algorithm. (a) Single regular obstacle. (b) Single irregular obstacle. (c) Multiple regular obstacles. (d) Multiple irregular obstacles.

**Fig 10:**
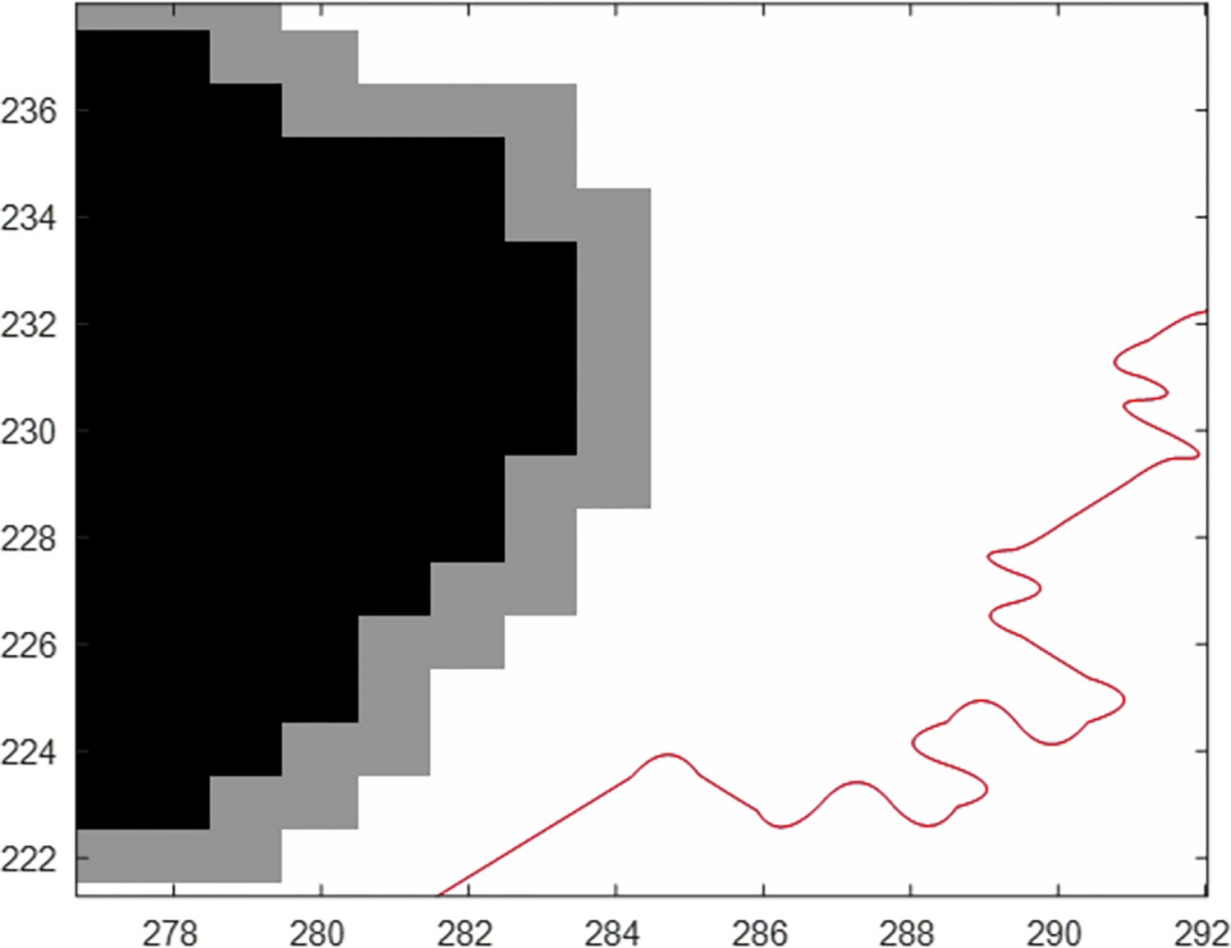
Multiple irregular obstacles enlarged the view of the planned path part of the VBAS algorithm.

To further verify the effectiveness of the VBAS algorithm, this paper selects another commonly used static path planning APF algorithm to study the path planning algorithm. The two algorithms were simulated multiple times on each map under the same operating environment to avoid chance, and the results are reported in Table 2. It can be seen from the results that although the path length obtained by VBAS is not better than that of APF, it has obvious advantages in time. Fig 9 shows the path planning results of the VBAS algorithm and APF algorithm in the same environment. Fig 10 is a partially enlarged view of the VBAS planned path in Fig 9D. Due to the size of the map, the APF planned path in Fig 8 is the same smoothness as the VBAS planned path.

#### 4.2.2 Comparison with traditional BAS algorithm

**Fig 11:**
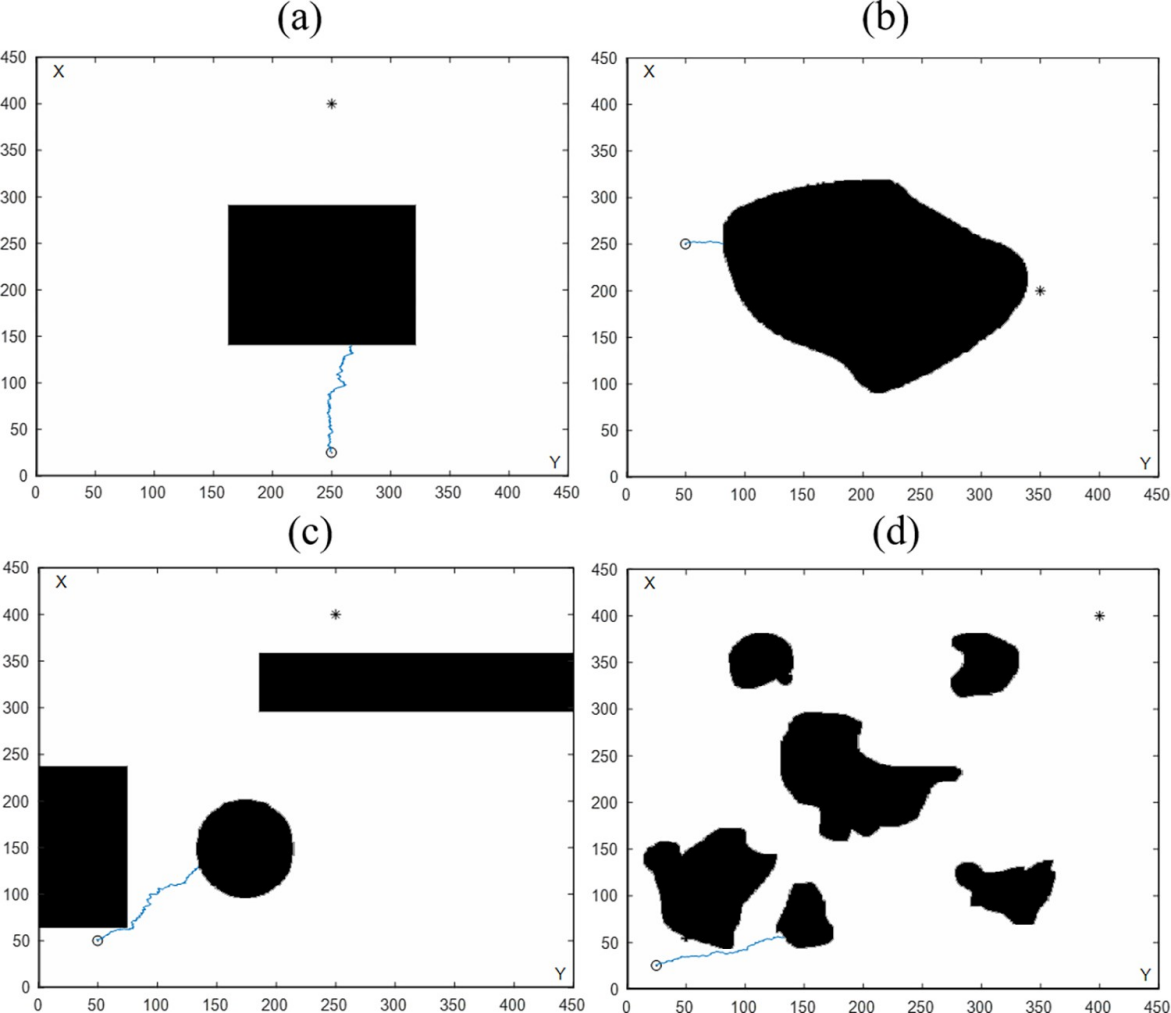
Comparison of path planning results by the conventional BAS algorithm on four different maps. (a) Single regular obstacle. (b) Single irregular obstacle. (c) Multiple regular obstacles. (d) Multiple irregular obstacles.

The effect diagram of VBAS is shown in Figs 5–8 to prove the effectiveness of the VBAS algorithm. It can be seen from Figs 5–8 that VBAS can effectively avoid obstacles and can effectively avoid obstacles when facing too large obstacles and irregular obstacles. The traditional BAS algorithm in Fig 11 does not introduce virtual target points in the simulation environment, so the vehicle cannot avoid obstacles and cannot reach the target point. By comparing VBAS and BAS algorithms, it is concluded that the VBAS algorithm can avoid obstacles, which is significantly improved.

## 5. Conclusion

This paper proposes an improved VBAS intelligent optimization algorithm based on the BAS algorithm. To verify the algorithm’s effectiveness, it applies to the path planning of vehicles. The specific case is to plan a collision-free and smooth path in a static environment under the premise of giving starting and target points. In addition, simulation results provide to verify the method’s effectiveness. In future work, the three-dimensional dynamic environment can replace the two-dimensional static environment, and the path planning research of the dynamic obstacle environment can be carried out in the 3D environment.

## Supporting information

S1 Dataset (XLSX)

## References

[1] Yi J B, Kang T, Song D, et al. Unified software platform for intelligent home service robots[J]. Applied Sciences, 2020, 10(17): 5874. doi: 10.3390/app10175874

[2] Yasuda Y D V, Martins L E G, Cappabianco F A M. Autonomous visual navigation for mobile robots: A systematic literature review[J]. ACM Computing Surveys (CSUR), 2020, 53(1): 1–34. doi: 10.1145/3368961

[3] Wang Y, Li X, Zhang J, et al. Review of wheeled mobile robot collision avoidance under unknown environment[J]. Science Progress, 2021, 104(3): 00368504211037771. 10.1177/00368504211037771. PMC10450763 34379021

[4] Verma J K, Ranga V. Multi-Robot Coordination Analysis, Taxonomy, Challenges and Future Scope[J]. Journal of intelligent & robotic systems, 2021, 102(1): 1–36. 10.1007/s10846-021-01378-2. PMC8051283 33879973

[5] Erke S, Bin D, Yiming N, et al. An improved A-Star based path planning algorithm for autonomous land vehicles[J]. International Journal of Advanced Robotic Systems, 2020, 17(5): 1729881420962263. 10.1177/1729881420962263.

[6] Zhang J, Wu J, Shen X, et al. Autonomous land vehicle path planning algorithm based on improved heuristic function of A-Star[J]. International Journal of Advanced Robotic Systems, 2021, 18(5): 17298814211042730. 10.1177/17298814211042730.

[7] Saravanakumar A, Kaviyarasu A, Ashly Jasmine R. Sampling based path planning algorithm for UAV collision avoidance[J]. Sādhanā, 2021, 46(3): 1–8. 10.1007/s12046-021-01642-z.

[8] Kwon H, Cha D, Seong J, et al. Trajectory Planner CDT-RRT* for Car-Like Mobile Robots toward Narrow and Cluttered Environments[J]. Sensors, 2021, 21(14): 4828. 10.3390/s21144828. 34300569 PMC8309725

[9] Quemelli M B, Brandão A S. Handling and pushing objects using unmanned guided vehicles[J]. Robotics and Computer-Integrated Manufacturing, 2020, 63: 101913. 10.1016/j.rcim.2019.101913.

[10] Zhu D D, Sun J Q. A New Algorithm Based on Dijkstra for Vehicle Path Planning Considering Intersection Attribute [J]. IEEE Access, 2021, 9: 19761–19775.

[11] Jayaweera H M, Hanoun S. A Dynamic Artificial Potential Field (D-APF) UAV Path Planning Technique for Following Ground Moving Targets[J]. IEEE Access, 2020, 8: 192760–192776.

[12] Lin Z, Yue M, Chen G, et al. Path planning of mobile robot with PSO-based APF and fuzzy-based DWA subject to moving obstacles [J]. Transactions of the Institute of Measurement and Control, 2022, 44(1): 121–132. 10.1177/01423312211024798.

[13] Wenzheng L, Junjun L, Shunli Y. An Improved Dijkstra’s Algorithm for Shortest Path Planning on 2D Grid Maps[C]//2019 IEEE 9th International Conference on Electronics Information and Emergency Communication (ICEIEC). IEEE, 2019: 438–441.

[14] Nie Z, Zhao H. Research on Robot Path Planning Based on Dijkstra and Ant Colony Optimization[C]//2019 International Conference on Intelligent Informatics and Biomedical Sciences (ICIIBMS). IEEE, 2019: 222–226.

[15] Zhang Z, Wu J, Dai J, et al. Optimal path planning with modified A-Star algorithm for stealth unmanned aerial vehicles in 3D network radar environment[J]. Proceedings of the Institution of Mechanical Engineers, Part G: Journal of Aerospace Engineering, 2022, 236(1): 72–81. 10.1177/09544100211007381.

[16] Hou X, Liu F, Wang R, et al. A UAV Dynamic Path Planning Algorithm[C]//2020 35th Youth Academic Annual Conference of Chinese Association of Automation (YAC). IEEE, 2020: 127–131.

[17] Miao C, Chen G, Yan C, et al. Path planning optimization of indoor mobile robot based on adaptive ant colony algorithm[J]. Computers & Industrial Engineering, 2021, 156: 107230. 10.1016/j.cie.2021.107230.

[18] Fu J, Lv T, Li B. Underwater Submarine Path Planning Based on Artificial Potential Field Ant Colony Algorithm and Velocity Obstacle Method[J]. Sensors, 2022, 22(10): 3652. 10.3390/s22103652. 35632060 PMC9146191

[19] Wang C, Wang Z, Zhang L, et al. Post-Impact Motion Planning and Tracking Control for Autonomous Vehicles[J]. Chinese Journal of Mechanical Engineering, 2022, 35(1): 1–18. 10.1186/s10033-022-00745-w.

[20] Zhang Z, Zhang L, Deng J, et al. An Enabling Trajectory Planning Scheme for Lane Change Collision Avoidance on Highways[J]. IEEE Transactions on Intelligent Vehicles, 2021.

[21] Jiang X, Lin Z, He T, et al. Optimal Path Finding With Beetle Antennae Search Algorithm by Using Ant Colony Optimization Initialization and Different Searching Strategies[J]. IEEE Access, 2020, 8: 15459–15471.

[22] Wu Q, Shen X, Jin Y, et al. Intelligent Beetle Antennae Search for UAV Sensing and Avoidance of Obstacles [J]. Sensors, 2019, 19(8): 1758. 10.3390/s19081758. PMC6514918 31013782

[23] Chen W J, Jhong B G, Chen M Y. Design of path planning and obstacle avoidance for a wheeled mobile robot[J]. International Journal of Fuzzy Systems, 2016, 18(6): 1080–1091. 10.1007/s40815-016-0224-7.

[24] Sun X, Liu Y, Yao W, et al. Triple‐stage path prediction algorithm for real‐time mission planning of multi‐UAV[J]. Electronics Letters, 2015, 51(19): 1490–1492. 10.1049/el.2015.1244.

[25] Wu Q, Ma Z, Xu G, et al. A novel neural network classifier using beetle antennae search algorithm for pattern classification[J]. IEEE Access, 2019, 7: 64686–64696.

[26] Eshtehardian S A, Khodaygan S. A continuous RRT*-based path planning method for non-holonomic mobile robots using B-spline curves[J]. Journal of Ambient Intelligence and Humanized Computing, 2022: 1–10. 10.1007/s12652-021-03625-8.

